# Radiation eye dose to medical staff during respiratory endoscopy under X-ray fluoroscopy

**DOI:** 10.1093/jrr/rraa034

**Published:** 2020-07-13

**Authors:** Yoshihiro Haga, Koichi Chida, Yuichiro Kimura, Shinsuke Yamanda, Masahiro Sota, Mitsuya Abe, Yuji Kaga, Taiichiro Meguro, Masayuki Zuguchi

**Affiliations:** 1 Department of Radiological Technology, Tohoku University Graduate School of Medicine, 2-1 Seiryo, Aoba, Sendai 980-8575, Japan; 2 Department of Radiology, Sendai Kousei Hospital, Sendai, Miyagi, Japan, Hirosemachi 4-15, Aobaku, Sendai 980-0873, Japan; 3 Division of Disaster Medicine, International Research Institute of Disaster Science, Tohoku University, 6-6-4, Aoba, Sendai 980-8579, Japan; 4 Department of Respiratory Medicine, Sendai Kousei Hospital, Sendai, Miyagi, Japan, Hirosemachi 4-15, Aobaku, Sendai 980-0873, Japan; 5 Department of Cardiovascular Medicine, Sendai Kousei Hospital, Sendai, Miyagi, Japan, Hirosemachi 4-15, Aobaku, Sendai 980-0873, Japan

**Keywords:** radiation safety eye lens dose, x-ray fluoroscopy, fluoroscopically guided respiratory endoscopy (bronchoscopy), dosimeter, radiation disaster medicine, 3-mm dose-equivalent [Hp(3)]

## Abstract

Although the clinical value of fluoroscopically guided respiratory endoscopy (bronchoscopy) is clear, there have been very few studies on the radiation dose received by staff during fluoroscopically guided bronchoscopy. The International Commission on Radiological Protection (ICRP) is suggesting reducing the occupational lens dose limit markedly from 150 to 20 mSv/year, averaged over defined periods of five years. The purpose of this study was to clarify the current occupational eye dose of bronchoscopy staff conducting fluoroscopically guided procedures. We measured the occupational eye doses (3-mm-dose equivalent, Hp(3)) of bronchoscopy staff (physicians and nurses) over a 6-month period. The eye doses of eight physicians and three nurses were recorded using a direct eye dosimeter, the DOSIRIS. We also estimated eye doses using personal dosimeters worn at the neck. The mean ± SD radiation eye doses (DOSIRIS) to physicians and nurses were 7.68 ± 5.27 and 2.41 ± 1.94 mSv/6 months, respectively. The new lens dose limit, 20 mSv/year, may be exceeded among bronchoscopy staff, especially physicians. The eye dose of bronchoscopy staff (both physicians and nurses) was underestimated when measured using a neck dosimeter. Hence, the occupational eye dose of bronchoscopy staff should be monitored. To reduce the occupational eye dose, we recommend that staff performing fluoroscopically guided bronchoscopy wear Pb glasses. correct evaluation of the lens dose [Hp(3)] using an eye dosimeter such as the DOSIRIS is necessary for bronchoscopy staff.

## INTRODUCTION

Lung cancer is one of the most common causes of cancer-related deaths in the world. Respiratory endoscopy (bronchoscopy) is one of the most practical modalities for obtaining lung tissue samples. With the technological advances in bronchoscopy, accurate evaluation of the tracheobronchial tree is now available. Hence, bronchoscopy has become very useful for diagnosis and therapeutic treatment of lung diseases, especially lung cancer [1–3].

Diagnostic and treatment outcomes for bronchoscopy may be improved by fluoroscopic guidance to target the areas of interest more accurately. In addition, using X-ray fluoroscopy during bronchoscopy can help prevent procedural complications such as pneumothorax [4,5].

Although the clinical value of fluoroscopically guided bronchoscopy is clear, there have been very few studies on the radiation dose received by staff during fluoroscopically guided bronchoscopy [6,7]. Furthermore, no report has discussed the occupational eye dose in bronchoscopy under fluoroscopy.

During fluoroscopically guided procedures such as interventional radiology, staff and patients may be injured by prolonged exposure to fluoroscopy X-ray radiation [8–15]. Currently, the International Commission on Radiological Protection (ICRP) is suggesting reducing the occupational lens dose limit markedly from 150 mSv per year to 20 mSv per year, averaged over defined periods of five years, with no annual dose in a single year exceeding 50 mSv [16,17]. Hence, it is essential to evaluate the medical occupational eye dose, including the radiation eye dose received by medical workers during bronchoscopy procedures.

The purpose of this study was to clarify the current occupational eye dose of bronchoscopy staff conducting fluoroscopically guided procedures, using a direct eye dosimeter. Furthermore, we compared the eye dose of bronchoscopy staff (physicians and nurses) measured directly using an eye dosimeter versus that measured using a personal dosimeter worn at the neck.

## MATERIALS AND METHODS

### Subjects

All procedures were conducted with patients in the supine position, and flexible bronchoscopy was performed using a fiberoptic bronchoscope (BF-P240, BF-260, BF-P260F, BF-P260F-OL8, Olympus, Japan). Each procedure was conducted under local anesthesia with conscious sedation, and the scope was inserted orally.

**Fig. 1.**
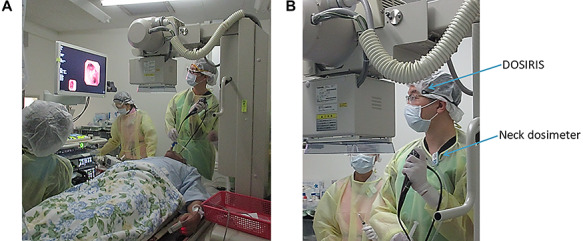
The members of the medical staff as they perform bronchoscopy. Note that they are wearing lead gowns for radiation protection. Over couch X-ray system was used for procedures. Usually, the source of scatter radiation (patient and X-ray tube housing) were close to the left side the main physician (operator). In contrast, the standing location and orientation of the nurses were inconsistent. b. The positions of the dosimeters used during the procedures. The eye dosimeter (DOSIRIS) was worn just lateral to the left eye, and the neck dosimeter (badge) was worn outside the Pb apron to the left of the neck.

This study was conducted at Sendai-Kosei Hospital over a 6-month period, from April to September 2018, during which 100 (mean) diagnostic bronchoscopy procedures were performed (Table 1). The occupational radiation exposure of the eyes (eye dose) of eight physicians and three nurses during bronchoscopy was measured using a dosimeter at 1-month intervals within this period. We also calculated the cumulative 6-month eye doses (half-year occupational dose) of bronchoscopy staff. During these bronchoscopy procedures, the staff always wore protective aprons (usually composed of a 0.35-mm Pb equivalent). Staff did not wear Pb glasses. We used an over-the-table X-ray fluoroscopy system (WINSCOPE^TM^20 DREX-WIN20, Toshiba, Japan) with an image intensifier (field size: 11 in) for all procedures (Fig. 1). Continuous X-ray fluoroscopy was used to guide insertion of the bronchoscope. The tube potentials (energies) of the x-ray fluoroscopes were approximately 80 kVp.

**Table 1:** Summary of our 6-month bronchoscopy study

	n	Neck badge, Hp(0.07), [mSv/6 months]	DOSIRIS (Left), Hp(3), [mSv/6 months]	Number of procedures
Physicians	8	5.55 (0.30-12.20)	7.68 (0.57-15.82)	100 (62-186)
Nurses	3	1.63 (0.10-2.62)	2.41 (0.19-3.76)	100 (97-102)

Average (Range, Min-Max)

In all procedures, the main physician was positioned close to the head of the patient. During our bronchoscopy procedures, the X-ray tube and patient were usually positioned close to the left side of the main physician. In contrast, the distance between the nurses and the patient was approximately 2 m, although some variation occurred.

This study was approved by the ethics committee of our institution (The ethics committee of the Sendai Kosei Hospital). Informed consent was obtained from all subjects. All bronchoscopy procedures were performed in accordance with the guidelines promulgated by the Japan Society for Respiratory Endoscopy [18].

### Dosimetry

The methods used to evaluate the eye radiation dose have been described previously [19]. Briefly, the staff participating in this study used a direct eye-lens dosimeter (eye dosimeter), the DOSIRIS (IRSN, France), which specifically measures the eye lens dose [3-mm-dose equivalent, Hp(3)] [20]. The eye dosimeter consists of a thermoluminescent dosimeter sensor (^7^LiF:Mg,Ti), a plastic capsule, and an adjustable headset. The laboratory at Chiyoda-Technol (Japan) supplied and calibrated the eye dosimeters. Following each 1-month measurement period, the eye dosimeters were returned to Chiyoda-Technol to be read. Dose calibration of the eye dosimeter was performed at Chiyoda-Technol using the slab phantom following Japanese Industrial Standards (JIS Z4345).

All staff wore an eye dosimeter just lateral to the left eye, and all nurses also wore an eye dosimeter (DOSIRIS) just lateral to the right eye (Fig. 1b). In addition, four physicians also wore an eye dosimeter (DOSIRIS) just lateral to the right eye for 3 months, from July 2018 to September 2018. Thus, the physicians wore an eye dosimeter (DOSIRIS) just lateral to the left eye, while four of them wore it lateral to the right eye additionally. The nurses wore them at the both sides.

We also used a commercial silver-activated phosphate glass personal dosimeter (70-μm-dose-equivalent, [Hp(0.07)], Glass Badge, Chiyoda-Technol), which was worn outside the Pb apron to the left of the neck, to estimate the eye doses of all staff. We measured the correlation between the eye [Hp(3)] and neck dosimeter doses [Hp(0.07)] to determine whether it is feasible to estimate the eye dose using a neck dosimeter, although Hp(3) and Hp(0.07) are different quantity in the definition.

We determined the estimated annual eye dose as follows:

Estimated annual eye dose (mSv/year) *=* dose measured over 6 months × 2

### Statistical analysis

The Wilcoxon signed-rank test was used to compare eye doses between two groups (neck-badge dose vs. eye-dosimeter dose, and left-side dose vs. right-side dose). Correlations between the neck and eye dosimeter measurements were analyzed by linear regression. Statistical significance was defined as *p*<0.05.

## RESULTS

Table 1 summarizes the results of our 6-month bronchoscopy study. The eye doses received by bronchoscopy staff were markedly higher in physicians than in nurses. The duration (mean ± SD) of fluoroscopy in our 6-month bronchoscopy study was 4.3 ± 3.5 min.

### Physician dose

The mean ± SD radiation doses to neck badges and DOSIRIS were 5.55 ± 4.15 and 7.68 ± 5.27 mSv/6 months, respectively.

**Fig. 2.**
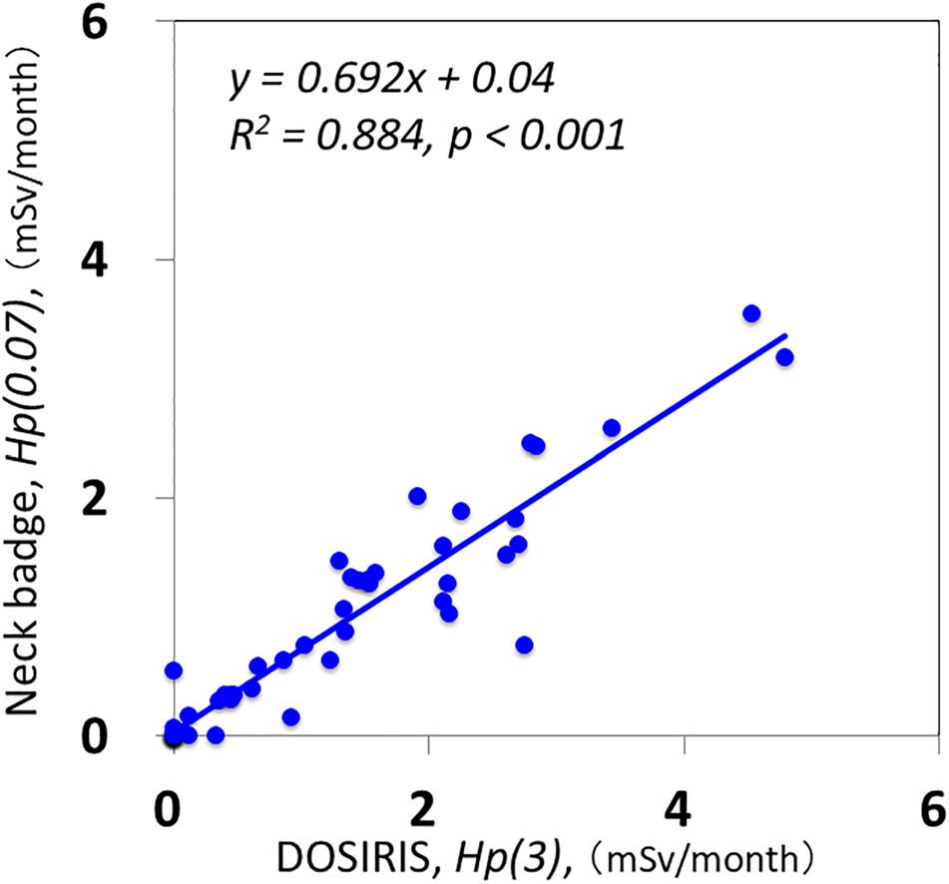
Relationship between eye (DOSIRIS) and neck dosimeter measurements (mSv/month) in eight physicians over 6 months. (n = 48)

**Fig. 3.**
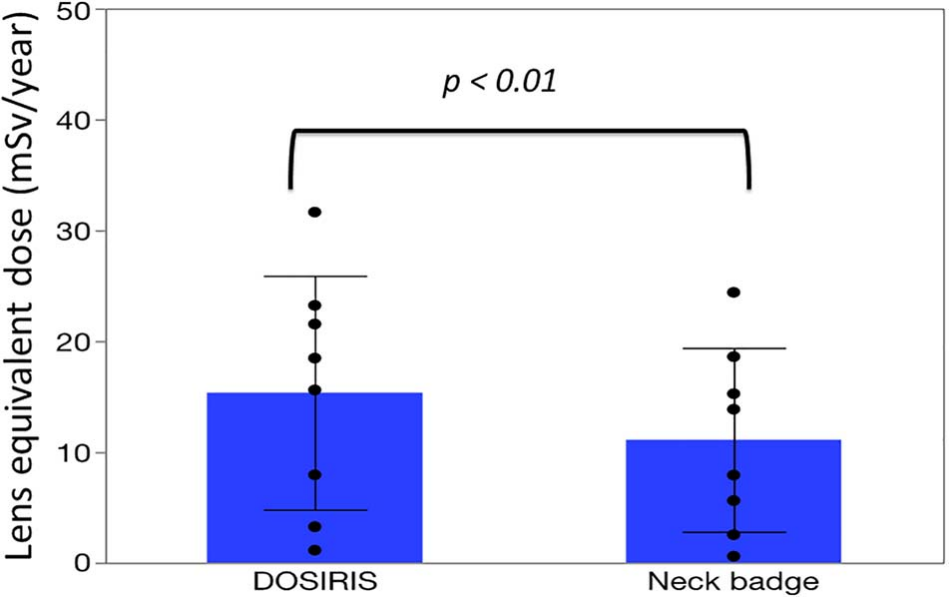
Mean ± SD estimated annual eye dose in eight physicians derived from eye (DOSIRIS) and neck badge dosimeter measurements. (n = 8). DOSIRIS, 15.4 ± 10.5; neck badge, 11.1 ± 8.3 (mSv/y).

**Fig. 4.**
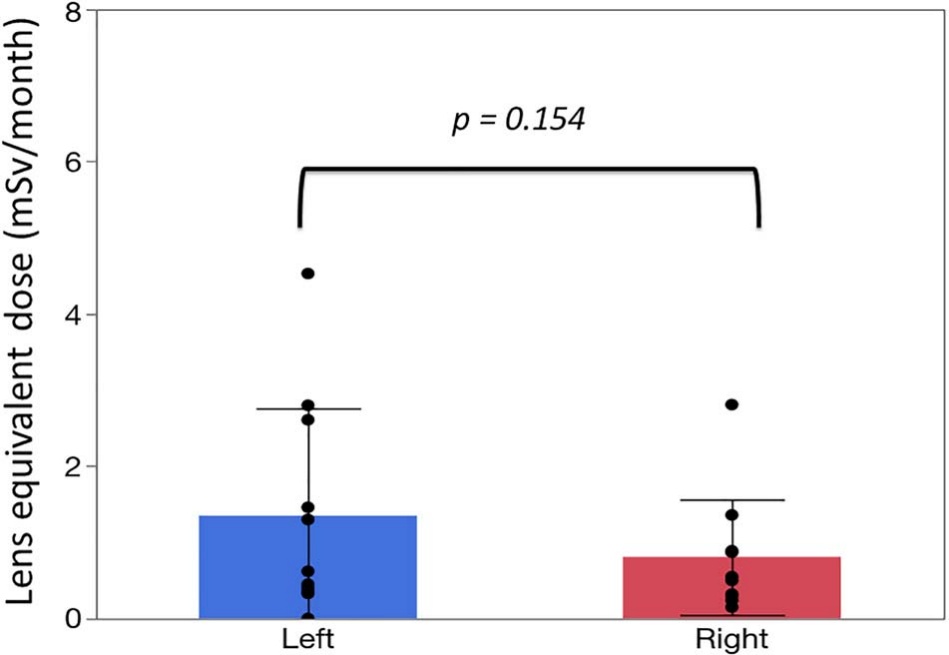
Mean ± SD monthly lens dose (left and right eyes) in four physicians derived from eye dosimeter (DOSIRIS) measurements over 3 months. (n=12). Left, 1.35 ± 1.41; Right, 0.81 ± 0.76 (mSv/month).

There were significant correlations between the neck and eye dosimeter monthly measurements (Fig. 2). The estimated annual eye doses are shown in Fig. 3. According to the DOSIRIS measurements, three physicians exceeded the equivalent dose limit for the lens (20 mSv/year). The neck dosimeter tended to underestimate the eye dose (Fig. 3). In addition, the eye doses of physicians were approximately 1.7 times higher in the left eye than in the right eye (Fig. 4), although this difference was not statistically significant.

### Nurse doses

The mean ± SD radiation doses to neck badges and DOSIRIS were 1.63 ± 1.34 and 2.41 ± 1.94 mSv/6 months, respectively.

**Fig. 5.**
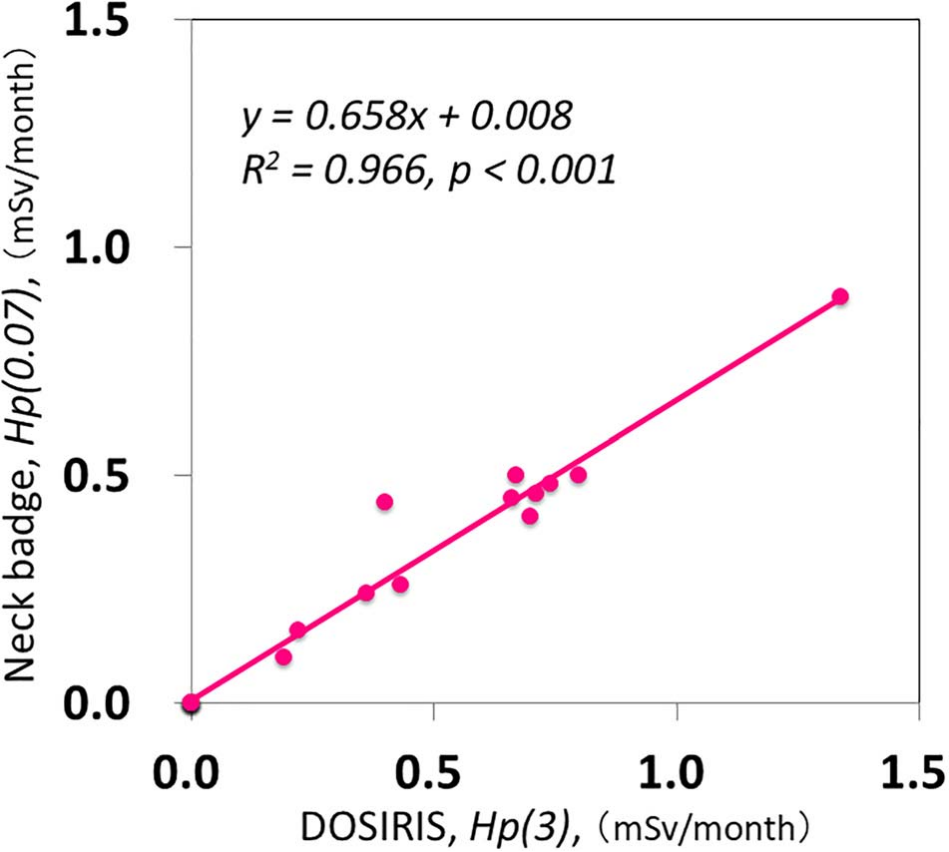
Relationship between eye (DOSIRIS) and neck dosimeter measurements (mSv/month) in three nurses over 6 months. (n= 18)

**Fig. 6.**
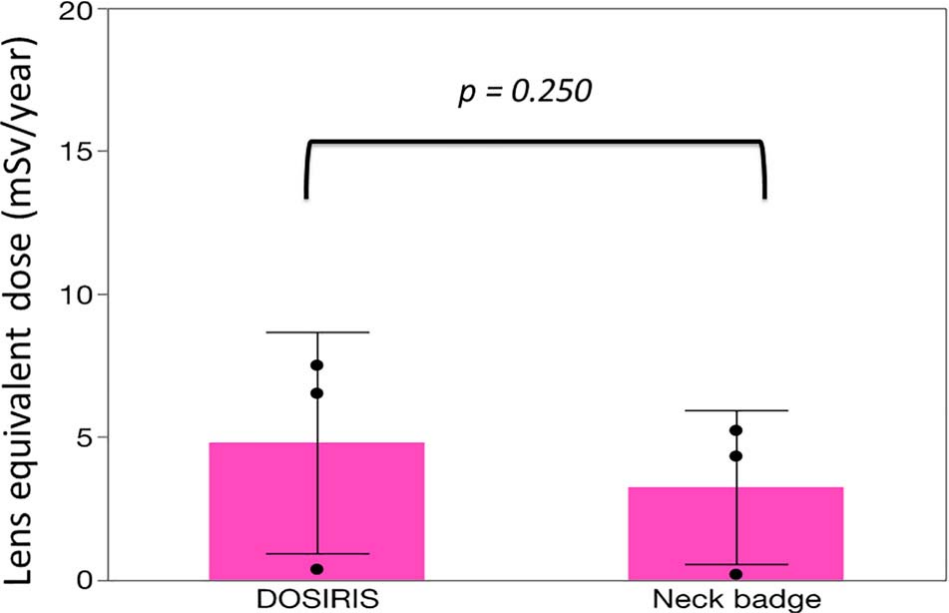
Mean ± SD estimated annual eye dose in three nurses derived from eye (DOSIRIS) and neck badge dosimeter measurements. DOSIRIS, 4.81 ± 3.87; neck badge, 3.26 ± 2.69 (mSv/y).

There were also significant correlations between the neck and eye dosimeter monthly measurements in nurses (Fig. 5). Figure 6 shows the estimated annual eye doses. No nurse exceeded the lens equivalent dose limit of 20 mSv/year. However, the occupational eye dose received by nurses cannot be ignored, and one nurse had an eye dose near 10 mSv/year; nurses may exceed the eye-dose limit if numerous procedures are performed.

**Fig. 7.**
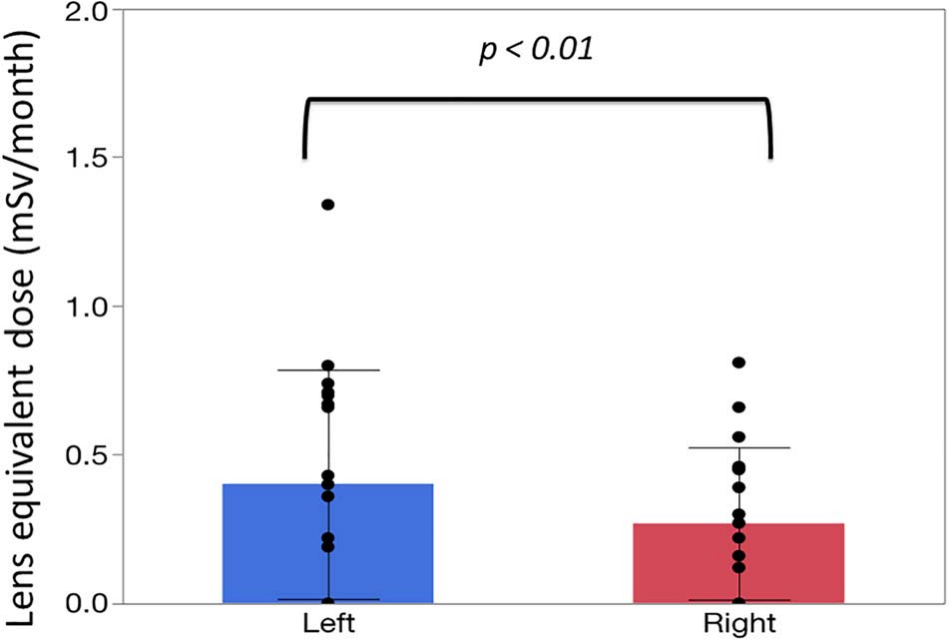
Mean ± SD monthly lens dose (left and right eyes) in three nurses derived from eye dosimeter (DOSIRIS) measurements. (n =18). Left, 0.40 ± 0.39; Right, 0.27 ± 0.26 (mSv/month).

The estimated annual eye dose in nurses evaluated using the neck dosimeter also tended to be underestimated compared with the eye dosimeter measurements (Fig. 6), although the difference was not significant. In addition, the eye doses were approximately 1.5 times higher in the left eye than in the right eye (Fig. 7).

## DISCUSSION

It is important to evaluate the medical occupational radiation eye dose [21–27]. This is the first detailed report on the occupational eye dose of staff (physicians and nurses) conducting fluoroscopically guided bronchoscopy.

In our study, the mean ± SD radiation eye doses (DOSIRIS) to physicians and nurses were 7.68 ± 5.27 and 2.41 ± 1.94 mSv/6 months, respectively. The occupational eye doses in bronchoscopy staff (physicians and nurses) are high, particularly in physicians, as well as in cardiac interventional radiology staff [19]. The eye dose in bronchoscopy physicians may exceed the new regulatory dose limit. Therefore, we recommend that bronchoscopy staff, especially physicians, should wear Pb glasses during procedures. In addition, evaluation of the shielding effect of Pb glasses during bronchoscopy procedures is necessary. We estimate that use of Pb glasses during bronchoscopy may afford shielding of about 50% [19,28,29].

We also recommend the use of additional shielding devices such as Pb acrylic shields suspended from the ceiling during bronchoscopy procedures, if available [30–32]. However, Pb acrylic shields suspended from the ceiling are inconvenient in clinical settings, and therefore manufacturers need to develop more ergonomically effective protection devices.

Bronchoscopy in Japan is usually conducted using an over-the-table (couch) X-ray tube system. When an over-the-table X-ray tube system (anterior-posterior, AP view) is used, the upper part of the staff’s body (including the eyes) receives high doses of scattered radiation [30,33]. Thus, to reduce the eye dose of bronchoscopy staff, use of an under-the-table X-ray tube system (PA [posterior-anterior] view) is preferred, if available.

We found that the radiation dose to the eyes of bronchoscopy staff tended to be underestimated using neck dosimeter measurements compared with direct measurement using the DOSIRIS. This may have been because the distance between the DOSIRIS and the X-ray tube housing is shorter than that between a neck badge and the X-ray tube. Hence, we recommend that bronchoscopy staff use an eye dosimeter such as the DOSIRIS for direct evaluation of eye dose.

Among the DOSIRIS measurements in bronchoscopy staff, the dose was higher in the left eye than in the right eye in our study. This may have been because the distance between the left DOSIRIS and the scattered radiation sources (the X-ray tube housing and patient) was smaller than that between the right DOSIRIS and these sources in our bronchoscopy procedures. Therefore, we recommend that staff wear an eye dosimeter on the side nearest the X-ray tube (the left side in our setup) during bronchoscopy procedures. Radiation safety education for bronchoscopy staff is important.

In summary, correct evaluation of the occupational eye radiation doses of medical staff is very important, including those performing bronchoscopy. We measured the occupational eye doses [Hp(3)] of bronchoscopy staff (physicians and nurses) over a 6-month period. The eye doses of eight physicians and three nurses were recorded using a direct eye dosimeter, the DOSIRIS. We also estimated eye doses using personal dosimeters worn at the neck. The eye dose of physicians may exceed the new regulatory occupational limit. Therefore, we recommend that bronchoscopy staff, especially physicians, wear Pb glasses during the procedures. The eye dose of bronchoscopy staff evaluated using neck dosimeters was underestimated compared with direct measurements using the DOSIRIS. Hence, we recommend that bronchoscopy staff use an eye dosimeter such as the DOSIRIS for correct evaluation of the lens dose. The staff doses measured were higher in the left eye than in the right eye.

## CONCLUSIONS

We evaluated the radiation eye dose of bronchoscopy staff performing X-ray fluoroscopy. This is the first report of the occupational eye dose of staff (physicians and nurses) performing fluoroscopically guided bronchoscopy. The new lens dose limit, 20 mSv/year, may be exceeded among bronchoscopy staff, especially physicians. Hence, the occupational eye dose of bronchoscopy staff should be monitored. To reduce the occupational eye dose, we recommend that staff performing fluoroscopically guided bronchoscopy wear Pb glasses.

The eye dose of bronchoscopy staff (both physicians and nurses) was underestimated when measured using a neck dosimeter. Correct evaluation of the lens dose [Hp(3)] using an eye dosimeter such as the DOSIRIS is necessary for bronchoscopy staff.

## CONFLICT OF INTEREST

Nothing.

## PRESENTATION AT A CONFERENCE

Nothing.

## CLINICAL TRIAL REGISTRATION NUMBER IF REQUIRED

Nothing.

## FUNDING

This study was supported in part by the Industrial Disease Clinical Research Grants (180501-1), Japan. This study also was supported in part by the Radiation Safety Research Promotion Fund, Nuclear Regulation Authority, Japan.
